# Lower ambulatory care availability and greater hospital capacity are associated with higher hospital case volumes

**DOI:** 10.1007/s43999-025-00066-0

**Published:** 2025-06-10

**Authors:** Doreen Müller, Manas K. Akmatov, Dominik Graf von Stillfried

**Affiliations:** 1 Center for Health and Human Sciences, Charité –Universitätsmedizin Berlin, Institute of Medical Sociology and Rehabilitation Science, Berlin, Germany; 2 Central Research Institute of Ambulatory Health Care, Berlin, Germany

**Keywords:** Hospital planning, Influences across care sectors, Healthcare supply factors, Geospatial analysis, Data linkage

## Abstract

**Introduction:**

The German hospital reform introduces population-based planning to allocate hospital budgets, considering each hospital’s role in meeting regional care needs. However, current hospital case numbers may reflect supply-side factors, such as physician density and socioeconomic disparities, rather than actual morbidity. Ambulatory care utilization inversely correlates with hospital usage, emphasizing the need to integrate ambulatory sector data into hospital planning. This study examines factors influencing hospital and office-based case numbers at the district level.

**Methods:**

Linking 2021 data from the Federal and State Statistical Offices, INKAR data and health insurance claims data in Germany at the district level, a multiple linear regression model assessed the association between case counts in hospitals or office-based practices per 10,000 residents and distance to the nearest general practitioner (GP), as well as hospital bed and GP density. The Global Moran’s I as well as a geographically weighted regression (GWR) analysis were conducted to assess regional differences.

**Results:**

Multiple linear regression revealed that greater GP distance, fewer GPs and more hospital beds were linked to more hospital cases, while office-based cases rose with shorter GP distance. Global Moran’s I confirmed spatial clustering, and GWR revealed heterogeneous effects of primary-care access on hospital admissions, whereas bed capacity uniformly increased hospital cases and shorter GP distances consistently predicted more office visits across Germany.

**Discussion:**

Our findings align with research showing supply-induced demand of hospital cases and emphasize the need for coordinated hospital and ambulatory care planning to improve access, reduce unnecessary hospital admissions, and optimize patient outcomes.

## Introduction

### Hospital financing in Germany before and after the reform

Until now, hospitals in Germany have been financed primarily through case-based reimbursements under the DRG (Diagnosis-Related Groups) system. This meant that hospitals received payment for each treatment case, the amount of which was determined by the diagnosis and the procedures performed. This created incentives to increase volume, because hospitals could only cover their costs or generate surpluses if they treated as many patients in profitable DRGs as possible. While average operating costs were refinanced via the DRG system, investment costs remained the responsibility of the federal states. Over time, the system was increasingly criticized for promoting volume driven care and exerting economic pressure on hospitals, rather than supporting needs based, regional health provision [1, 2].

In the course of the hospital reform in Germany (Hospital Care Improvement Act – KHVVG), which came into force on January 1, 2025, hospitals will receive so called standby funding to ensure the availability of essential services even when annual case volume per hospital decreases [3]. This marks a paradigm shift from volume oriented to structure oriented financing, with the goal of creating planning security and maintaining care capacities independent of case numbers. To qualify for funding, hospitals are assigned to initially 65 service groups based on the scope and quality of the services they offered during a reference period and their expected future role in providing inpatient care for a defined region. Compliance with structural and quality criteria defined for each service group is a prerequisite.

Another central element of the reform is the introduction of population-based planning approaches to better align hospital capacities with regional care needs. Hospital services will be planned and resources allocated based on the expected inpatient care demand in a region, rather than solely based on past case volumes. The explicit aim is to ensure comprehensive and equitable healthcare for all patients, including in rural areas. Hospitals must be reachable within a legally defined distance, depending on the service group, within a maximum driving time of 30 or 40 min. Implementation of these provisions in regional hospital planning is the responsibility of the 16 federal states.

In addition, the reform envisions that hospitals – particularly those designated as 'guarantee hospitals' – will offer outpatient specialist care in areas lacking resident specialists, which primarily affects rural regions.

### The role of hospital case volumes for financing and service structure of hospitals

With the reform, scheduled to be implemented by 2026, the role of hospital case volumes is changing. Whereas they were previously the central metric for hospital reimbursement, they are now expected, alongside other indicators, to play a role in assessing regional care needs and assigning hospitals to service groups. This means, on the one hand, that a higher number of hospital cases could be associated with greater care demand. At the same time, hospitals are to be present especially in regions with low healthcare facility density to provide outpatient specialist services locally, particularly in rural areas; sections where case volumes would, conversely, be lower.

### Which regional factors are associated with hospital case volumes?

To enable valid planning, health policy decision makers must therefore clarify to what extent hospital case volumes reflect actual population based care needs, or whether they are instead driven by supply side dynamics [4]. Regions with higher densities of physicians or hospitals often show higher utilization of health services due to easier and quicker access. More appointments are available, and patients can switch more readily among providers; overall, more patients can be treated [5]. This is mostly the case in urban regions. However, in regions with few doctors, there is naturally a cut-off limit to treatable cases. Even though in rural areas primary care physicians work more hours than their urban counterparts [6], case numbers are constrained by the very nature of workable hours per physician – in other words, by the supply structure. This may limit the extent of supply-induced demand in rural regions simply because the infrastructure for higher service volumes is lacking.

Socioeconomic factors also correlate with the supply structure: regions with good medical care are often socioeconomically advantaged [7] and tend to have lower morbidity rates [8]. Simultaneously, these regions are typically more urban, have higher population densities, and a younger age structure [9]. If actual morbidity alone determined case numbers, one would expect higher regional healthcare density and correlating healthier populations to coincide with lower relative case numbers.

### Aim of the present study

The aim of this study is, firstly, to examine the extent to which outpatient and inpatient supply structures are associated with hospital case numbers. It has been shown that better accessible outpatient structures lead to fewer hospital treatments [10]. Conversely, increased hospital capacity might induce demand, resulting in a higher relative number of hospital cases. To compare the two sectors of office-based and hospital treatment, we also investigate the statistical influence of office-based and hospital supply structures on office-based case numbers. This allows us to trace how office-based and hospital supply structures mutually influence one another. Additionally, due to regional specificities, these relationships may vary across different regions.

Our research questions are therefore:

1. Are relative hospital case numbers per region statistically influenced by indicators of office-based and hospital supply structure?
2. Are relative office-based case numbers per region statistically influenced by indicators of office-based and hospital supply structure?
3. Are these associations consistent across regions?

## Methods

### Study population and data sources

For this ecological study, three data sources were linked for the observation year 2021, as this is the most recent year for which all relevant indicators were available at the time of analysis. These sources include (A) the Regional Database (RDB) of the Federal and State Statistical Offices, (B) the Indicators and Maps for Spatial and Urban Development (INKAR) from the Federal Institute for Research on Building, Urban Affairs, and Spatial Development [11], as well as (C) nationwide ambulatory claims data, reported by physicians for billing purposes pursuant to §295 SGB V (Social Code Book V), covering individuals with statutory health insurance, which represent approximately 90% of the German population. The study population encompasses for data source (A) individuals with inpatient hospital cases (as recorded in the RDB), for data source (B) the entire population of Germany (as represented in the INKAR dataset), and for data source (C) individuals with statutory health insurance who visited an office-based doctor at least once in 2021.

Based on the territorial status as of December 31, 2021, Germany is divided into 400 rural and urban districts. These are the primary administrative units for local governance corresponding to the level 3 of the ‘Nomenclature des unites territoriales statistiques’ (NUTS-3) of the European Union. The districts serve as the basis for planning and providing regional services, including healthcare supply structures. This division into districts in our analysis allows for detailed regional comparisons of healthcare access and utilization patterns. Therefore, the three data sources were linked and analyzed at the district level, using individuals´ place of residence.

### Measures

For the outcome variables, the number of hospital cases and the number of office-based cases were selected. The data were aggregated for the year 2021 at the district level and then related to the respective population size. Therefore, both variables refer to the number of cases per 10,000 inhabitants in each district.

The explanatory variables for the office-based care structure were selected to capture key aspects of accessibility to ambulatory care. The average distance to the nearest general practitioner (GP) in meters per district provides a direct measure of geographic accessibility, which is critical in areas with varying population densities and distances to healthcare providers [12]. The number of GPs per 10,000 inhabitants was chosen as a proxy for the density of ambulatory care providers, which is a common measure of ambulatory healthcare access [7]. Both variables are important for understanding how proximity and availability of GPs impact access to health care services. Since the distance variable was only available for GPs and not for other types of office-based physicians, we opted to limit the density variable to GPs as well. This decision ensured consistency in the operationalization of the variables, avoiding potential confounding caused by including a wider range of office-based specialists whose distribution might differ from that of GPs.

For the hospital care structure, the number of hospital beds per 10,000 inhabitants was chosen as it reflects the availability of hospital services at the district level [13]. Hospital bed density is a widely recognized measure for assessing the capacity of hospital-based care and is particularly relevant when considering regional disparities in healthcare provision.

To account for demographic and socioeconomic differences between districts, we included variables that are known to influence both healthcare utilization and outcomes. The average age [14], proportion of women [15], and median income [16] were included to control for demographic and economic factors that could affect healthcare needs and access. The proportion of people in need of long-term care was also considered, serving as a proxy for the overall health status of a district's population, and influencing both ambulatory and hospital care demand [17].

### Statistical analysis

A multiple linear regression model was applied to the two research questions 1 and 2, resulting in two separate models with the outcome variable `number of hospital cases` for research question 1 and `number of office-based cases` for research question 2. Explanatory variables included the indicators of ambulatory and inpatient care supply as well as the selected sociodemographic and socioeconomic control variables. The significance level was set at 5%, and to account for multiple testing, p-values were adjusted using the Bonferroni-Holm method. All variables were checked for distribution and outliers prior to analysis. Multicollinearity among predictors was assessed using the variance inflation factor (VIF). Regression assumptions (normality, linearity, homoscedasticity) were evaluated through diagnostic plots. To ensure the interpretability of the regression models, the variables were not standardized.

To address research question 3, as a first step we computed the Global Moran’s I to assess whether the regions exhibited spatial autocorrelation in the outcome variables. Global Moran’s I is a measure of overall spatial autocorrelation, assessing whether similar values are clustered in space more than would occur by chance. It yields a statistic from −1 (indicating dispersion) through 0 (randomness) to + 1 (strong clustering), with significance testing to determine if the observed pattern departs from randomness [18]. In addition, we performed an exploratory geographically weighted regression (GWR) analysis to investigate how associations varied across regions [19]. GWR is a local extension of global linear regression that allows for the estimation of location-specific regression equations for each spatial unit (in this case, the 400 districts). Spatial weighting is applied using a kernel function that accounts for the distance between neighboring districts, assigning greater weight to those in closer proximity. The significance level was set at 5%; no correction for multiple testing was applied due to the exploratory nature of the analysis. Data preparation and analysis were conducted using Oracle SQL for data linkage and preprocessing, and R (version 4.4.1) for statistical analysis and visualization.

## Results

### Descriptives

**Fig. 1:**
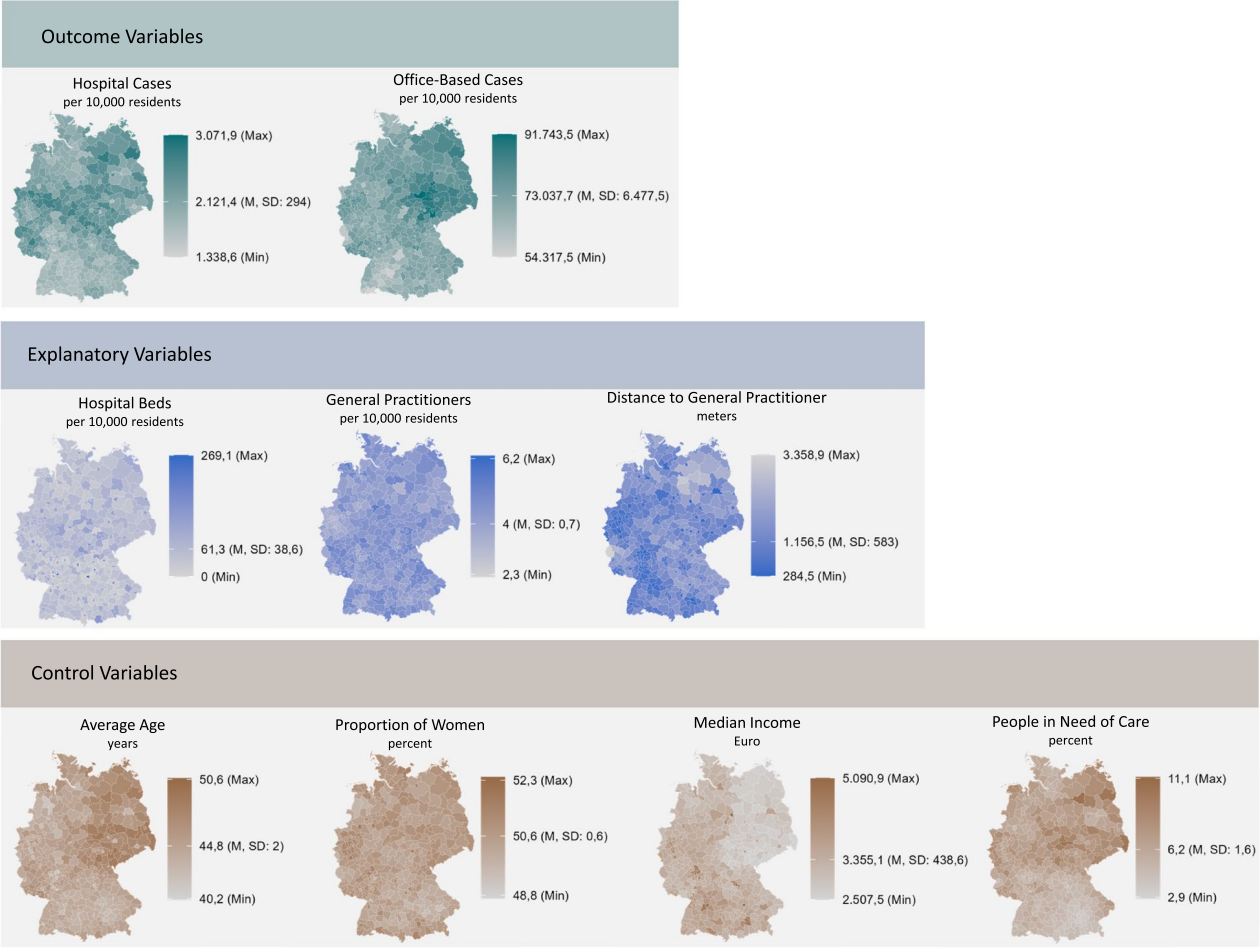
Descriptive presentation of outcome, explanatory, and control variables at the district level, 2021 (*N* = 400). *Legend: Max* = *maximum, M* = *mean, SD* = *standard deviation, Min* = *minimum*

In 2021, the relative hospital case rate ranged from 1,339 to 3,072 per 10,000 residents (mean: 2,121), with the highest rates observed in northeastern, central, and southwestern Germany. Office-based cases averaged 73,038 per 10,000 residents (range: 54,318–91,744), with concentrations predominantly in central Germany. Hospital beds per 10,000 residents exhibited a pronounced urban–rural gradient, with rural areas having significantly fewer beds. In contrast, the density of GPs per 10,000 residents varied little between urban and rural settings, although the average distance to the nearest practitioner was shortest in cities and in western Germany. For a comprehensive overview of all descriptive statistics, see Fig. 1.

Before conducting the regression analyses, all variables were thoroughly checked for distribution and outliers. Multicollinearity among predictors was assessed using the variance inflation factor (VIF), and no issues with multicollinearity were detected. Regression assumptions, including normality, linearity, and homoscedasticity, were evaluated through diagnostic plots. The residuals displayed no significant deviations from normality, and the scatterplots indicated a linear relationship between the predictors and the outcome variables. Additionally, the residuals versus fitted plot showed no signs of heteroskedasticity, with a uniform spread across the range of fitted values. These findings suggest that the assumptions for the linear regression models were met.

#### Research question 1: Are relative hospital case numbers per region statistically influenced by indicators of outpatient and inpatient supply structure?

The multiple linear regression model (see Table 1, section ‘Global Coefficient Estimates’) indicated that regional differences in the number of hospital cases were statistically associated with various indicators of the availability of medical services. A greater distance to the GP and a lower number of GPs were associated with more hospital cases at the district level (β_A.1_ = 0.09; 95% CI: [0.04; 0.13], *p* = 0.005; β_A.2_ = −51.25 [−82.64; −19.86], *p* = 0.005, respectively). Additionally, a higher number of hospital beds was significantly linked to an increased number of hospital cases (β_A.3_ = 1.36 [0.78; 1.94], *p* < 0.001). The model explained 57.4% of the regional variance of hospital case volumes (R^2^ = 0.574).

**Table 1:** Global and local coefficient estimates

	**Global** **Coefficient Estimates**^**§**^ **(Linear Regression)**			**Summary of Local Coefficient Estimates**^**§**^ **(Geographically Weighted Regression)**				
**Number of hospital cases per 10,000 residents**								
	**Estimate**	**95% Confidence Interval**	***p***value^**ǂ**^	**Minimum**	**1 st Quartile**	**Median**	**3rd Quartile**	**Maximum**
Intercept	726.39	−1247.05; 2699.83	1.000	−8,163.85	−399.06	1,176.77	3,059.25	11,226.82
Distance to general practitioner (meters)	0.09	0.04; 0.13	0.005	−0.46	0.00	0.08	0.14	0.28
General practitioners (per 10,000 residents)	−51.25	−82.64; −19.86	0.005	−134.20	−67.83	−24.01	3.74	105.26
Hospital beds (per 10,000 residents)	1.36	0.78; 1.94	0.000	0.16	0.93	1.55	2.38	7.88
Average age (years)	38.47	21.85; 55.10	0.000	−13.22	47.48	67.55	80.55	184.93
Proportion of women (percent)	−15.15	−54.75; 24.45	1.000	−313.06	−72.88	−36.57	5.64	118.52
Median income (Euro)	−0.02	−0.09; 0.05	1.000	−0.44	−0.12	−0.05	0.04	0.22
Proportion of people in need of care (percent)	85.90	68.03; 103.78	0.000	−51.86	40.52	51.01	76.39	198.27
*R-squared*	*0.574*					*0.837*		
**Number of office-based cases per 10,000 residents**								
	**Estimate**	**95% Confidence Interval**	***p***value^**ǂ**^	**Minimum**	**1 st Quartile**	**Median**	**3rd Quartile**	**Maximum**
Intercept	79,718.95	30,968.46; 128,469.43	0.004	−87,555.44	53,314.65	84,225.54	122,690.39	265,483.78
β_B.1_ Distance to general practitioner (meters)	−4.07	−5.3; −2.84	0.000	−9.16	−5.86	−4.29	−3.00	−1.30
β_B.2_ General practitioners (per 10,000 residents)	128.03	−647.44; 903.49	0.746	−2,456.83	−70.16	815.52	1,596.34	3,527.41
β_B.3_ Hospital beds (per 10,000 residents)	−10.49	−24.78; 3.8	0.300	−63.64	−34.84	−20.06	−5.10	30.93
Average age (years)	1,198.57	787.86; 1609.28	0.000	−1,326.08	649.18	1,118.79	1,426.74	2,846.38
Proportion of women (percent)	−1,048.04	−2026.34; −69.74	0.108	−5,245.79	−2,043.37	−964.29	−392.28	2,531.84
Median income (Euro)	−3.23	−4.88; −1.59	0.000	−7.22	−2.71	−1.25	0.56	9.21
Proportion of people in need of care (percent)	1,347.92	906.38; 1789.45	0.000	−3,592.80	518.73	1,351.18	1,970.09	3,664.06
*R-squared*	*0.466*					*0.736*		

*Data source: Hospital Cases per 10,000 Residents: RDB; Ambulatory Cases per 10,000 Inhabitants: billing data from statutory health insurance according to §295 SGB-V; all other variables: INKAR*

*Example interpretation for hospital cases, global coefficient estimates (for significant explanatory variables only): Each additional meter in average distance to the GP is associated with 0.09 more hospital cases per 10,000 residents. Each additional GP per 10,000 inhabitants is associated with 51.25 fewer hospital cases per 10,000 residents. For each additional hospital bed per 10,000 residents, there are 1.36 more hospital cases per 10,000 residents. The model explains 57.4% of the regional variation in hospital case numbers at the district level (R-squared)*

*Example interpretation for hospital cases, local coefficient estimates (for ‘Distance to general practitioner (meters)’ only): At the local level, the association between varies across the 400 regions. In some areas, the relationship is negative, with each additional meter in average distance to the GP being associated with 0.46 fewer (minimum value) or with up to 0.28 more (maximum value) hospital cases per 10,000 residents. The central tendency across the 400 local coefficients suggests that for each additional meter in average distance to the nearest GP, there is an associated increase of approximately 0.08 hospital cases per 10,000 residents (median value)*

*Example interpretation for office-based cases, global coefficient estimates (for significant explanatory variables only): Each additional meter in average distance to the GP is associated with 4.07 fewer hospital cases per 10,000 residents. The model explains 46.6% of the regional variation in hospital case numbers at the district level (R-squared)*

*Example interpretation for office-based cases, local coefficient estimates (for ‘Distance to general practitioner (meters)’ only): At the local level, the association varies across the 400 regions. In all areas, the relationship is negative, with each additional meter in average distance to the GP being associated with 9.16 (minimum value) to 1.30 (maximum value) fewer office-based cases per 10,000 residents. The central tendency across the 400 local coefficients suggests that for each additional meter in average distance to the nearest GP, there is an associated decrease of approximately 4.29 hospital cases per 10,000 residents (median value)*

^**§**^Adjusted for all variables in the table

^**ǂ**^Corrected for multiple testing

#### Research question 2: Are relative office-based case volumes per region statistically influenced by indicators of outpatient and inpatient supply structure?

Building on the previous analysis, the multiple linear regression model for office-based cases per 10,000 residents revealed a distinct pattern of associations (see Table 1, section ‘Global Coefficient Estimates’). Unlike hospital cases, a greater mean distance to the GP predicted significantly fewer office-based consultations (β_B.1_ = –4.07; 95% CI: [–5.30; –2.84], *p* < 0.001), whereas neither the density of GPs nor the number of hospital beds reached statistical significance. Overall, this model explained 46.6% of the variance in office-based case rates (R^2^ = 0.466).

#### Research question 3: Are these associations consistent across regions?

The Global Moran’s I for hospital cases (I = 0.631, *p* < 0.001) and for office-based cases (I = 0.628, *p* < 0.001) indicates a strong, statistically significant positive spatial autocorrelation, suggesting that regions with similarly high or low case counts tend to cluster rather than being randomly distributed.

**Fig. 2:**
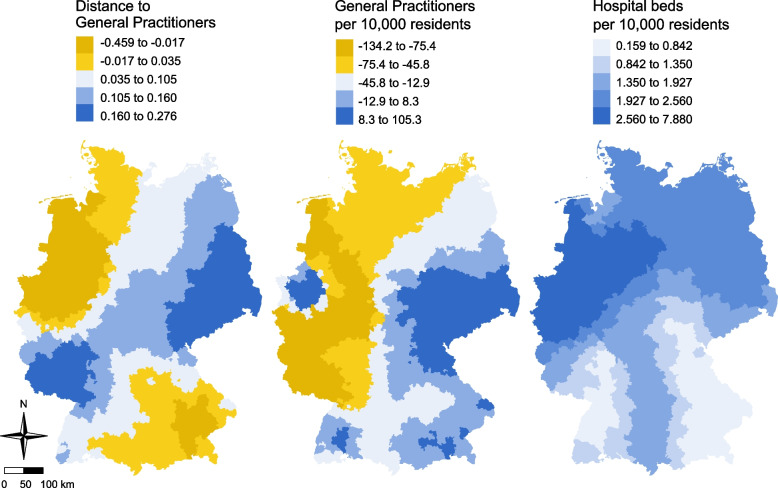
Local coefficient estimates for 2021: hospital case counts across 400 German districts – results of geographically weighted regression (GWR) analysis. Maps display the spatial distribution of local coefficients for the three predictors with significant global associations: distance to the nearest GP in meters, GP density per 10,000 residents, and hospital beds per district

The exploratory GWR results (Table 1, section ‘Summary of Local Coefficient Estimates’) reveal marked spatial heterogeneity in how primary‐care access and hospital capacity relate to hospital case counts. For distance to the nearest GP, the local regression coefficients ranged from −0.46 to + 0.28 (median: + 0.08), indicating that greater travel distance to the nearest GP was not consistently associated with more hospital cases across all districts. Likewise, the effect of GP density on hospitalizations varied between −134.20 and + 105.26 (median: −24.01), underscoring that lower GP availability did not uniformly predict higher admission rates. As shown in Fig. 2, both predictors exhibited predominantly negative associations in western Germany and positive associations in the east part of Germany. In contrast, the number of hospital beds per district was positively linked to case counts throughout Germany (coefficients: 0.16 to 7.88; median: 1.55), with the strongest effects observed in western and eastern regions and comparatively weaker associations in southern Germany.

**Fig. 3:**
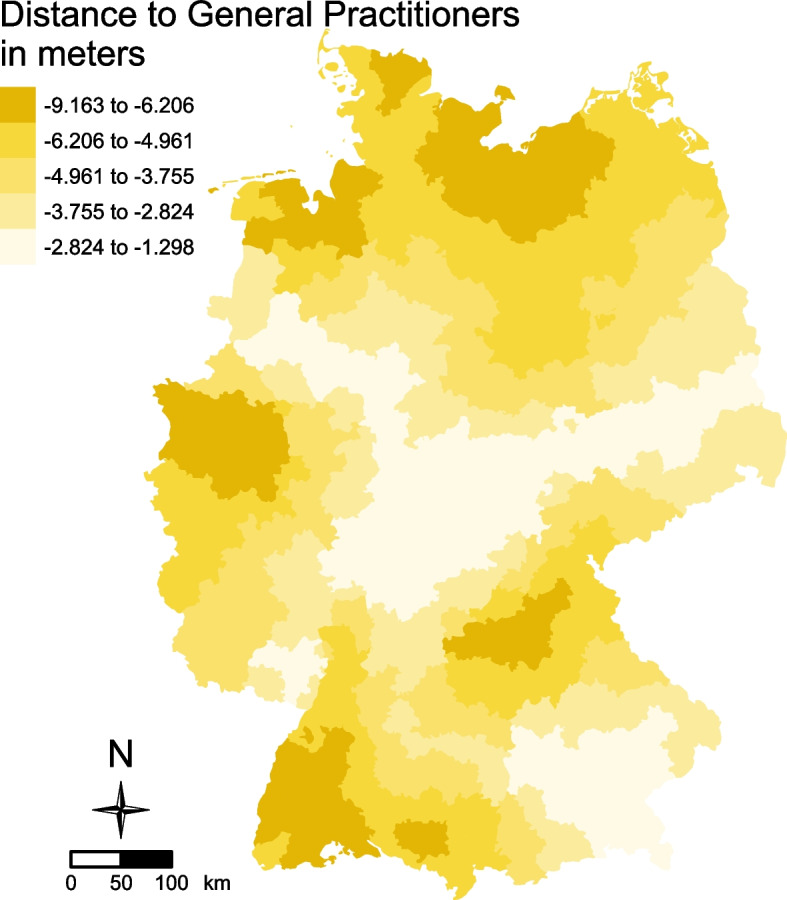
Local coefficient estimates for 2021: office-based case counts across 400 German districts – results of geographically weighted regression (GWR) analysis. Maps display the spatial distribution of local coefficients for the predictor with significant global association: distance to the nearest GP in meters

For office-based cases in the exploratory GWR, shorter distances to the nearest GP were uniformly linked to higher office-based visit counts, with local coefficients ranging from −9.16 to −1.30 (median: −4.29). Figure 3 reveals no clear regional trend, although small clusters of stronger associations emerge in the northern, western, and southwestern parts of Germany.

## Discussion

The present study aimed at identifying regional determinants of case counts in hospitals and office-based practices by linking utilization data with indicators of healthcare service availability on the district level, controlling for demographic and socioeconomic structure.

### Are the numbers of hospital and outpatient cases statistically influenced by supply structure?

The present analysis demonstrated that the number of hospital cases at the district level was higher when there were more hospital beds and fewer GPs relative to the population size, which is also reflected in previous research [20, 21]. Additionally, a greater distance to the nearest GP was associated with a higher number of hospital cases, a finding that has also been reported before [22]. The present findings can be contextualized within the existing research on supply-induced demand. It is shown that hospital case numbers particularly fluctuate regionally primarily with the hospital supply structure but are also affected by accessibility of ambulatory care [23, 24]. Additionally, in a number of previous studies, an inverse relationship between density of ambulatory specialty physicians and the number of (avoidable) inpatient cases for the resident population has been shown [25, 26]. On the other hand, office-based case numbers were unaffected by GP or hospital bed density, but proximity to the nearest GP was positively associated with case volume, which is in line with previous research [27].

On the global level, in regions with a well-developed hospital infrastructure but a limited supply of GPs, the number of hospital cases is relatively higher compared to regions with fewer hospitals and/or more GPs. In contrast, the number of office-based cases appears to be less influenced by the local supply structure. This suggests that a higher number of hospital cases does not necessarily reflect higher morbidity or need structure. Rather, some of these cases might have otherwise been treated in the ambulatory sector if sufficient outpatient care had been available. Greater distances to the nearest GP may pose a barrier to accessing preventive or timely curative care from an outpatient physician. It is possible that a lack of accessible outpatient care leads to delayed treatment, causing patients to enter the healthcare system only once their condition has worsened and hospital care becomes necessary [28, 29].

### Are these associations consistent across regions?

Across all regions, hospital bed availability showed a consistent positive association with hospital case numbers: districts with more beds per capita experienced higher case counts. However, the impact of primary-care access on hospital admissions varied regionally: although the global model linked greater distance to the nearest GP with higher hospitalization rates, the exploratory GWR revealed the opposite pattern in northwestern and southeastern Germany. One possible explanation is that more urbanized regions host a higher concentration of medical care centers (MVZ) [30], creating a denser primary-care network that may bias the observed relationship between hospital case numbers and GP distance. Additionally, inverse relationships might arise due to spill-over effects, where the number of cases per residential population exceeds what would be expected based on local care needs alone [24].

Contrary to the overall trend – where higher GP density predicted fewer hospital admissions – in the south of Eastern Germany greater numbers of GPs were actually associated with increased hospitalizations. This could be due to morbidity patterns, with East Germany having overall higher morbidity compared to West Germany [31–33], even though the gap is closing [34]. Furthermore, Saxony in particular experiences cross-border patient flows from the Czech Republic and Poland to some degree, meaning local providers serve a mixed patient population, and those with more complex needs may be hospitalized more frequently [35, 36].

A higher number of hospital beds consistently predicted a greater number of hospital cases across Germany, indicating that hospital utilization may be influenced by, alongside medical need, the available supply structure.

### Implications for the hospital reform

The results suggest that hospital case numbers reflect, at least in part, supply-induced demand rather than morbidity [21]. Therefore, when determining the necessary hospital density and locations, the number of hospital cases should be considered with caution. Financing entire hospitals to secure outpatient care may additionally not be the most effective and efficient approach. Beyond the higher treatment costs in the inpatient sector, other negative consequences can arise. For instance, hospital stays are reported to increase the likelihood of subsequent readmissions [28], raise mortality rates [29], and negatively impact frailty or cause delirium [37]. Furthermore, the frequency of ambulatory sensitive hospital cases is negatively associated with the density of resident physicians, and this relationship is particularly pronounced in rural regions [26]. This suggests that in regions where fewer outpatient physicians practice relative to the population, people are more often treated by specialist care [38] or in hospitals. If hospitals were to take over outpatient provision, they could further displace outpatient providers, with all the negative consequences that might follow.

Moreover, having a primary care physician has been linked with beneficial effects for both patient satisfaction and medical outcomes. Studies have shown that care through a primary care provider can lead to improved health outcomes, such as better management of chronic diseases, more preventative care, and higher patient satisfaction levels​ [39]. Also, continuity of care provided by primary care physicians is associated with higher patient satisfaction, lower mortality, lower costs and less hospitalizations [40, 41]. The relationship with a primary care doctor often leads to better communication and more personalized care, which are key factors for improving overall health and patient experience.

According to the reform, cross sectoral care facilities at hospitals and so called ´guarantee hospitals’ will in future also provide outpatient specialist services in regions where no resident specialists are active, predominantly rural areas. However, it remains to be seen whether this measure will bear fruit. The physician shortage in rural areas affects both outpatient and inpatient providers equally, as hospitals in sparsely populated regions also struggle to recruit suitable specialist staff [42, 43]. Strengthening office-based physicians in less densely populated regions may represent a more effective strategy to address healthcare challenges, as a stronger outpatient sector was associated with reduced use of hospital resources.

In conclusion, there is evidence that sufficient outpatient supply leads to reduced need for inpatient resources, meaning that regions with a higher density of office-based physicians might need less hospital resources. Accordingly, the number of outpatient service providers should be considered in future hospital planning to ensure the positive financial and health related outcomes of a predominantly outpatient approach that is in line with a public health orientation. Given that it is a defined aim of the hospital reform to reduce hospital overcapacities, ideally, hospital planning and ambulatory planning should go hand in hand to ensure a more integrated, efficient healthcare system that reduces unnecessary hospital admissions and optimizes patient outcomes. Coordinated planning between these sectors can improve access to care, enhance the continuity of treatment, and help balance healthcare resources, ultimately benefiting both patients and healthcare providers and leading to lower healthcare costs as long as priority is given to ambulatory care wherever possible.

### Methodological considerations and outlook

The strengths of the present analyses lie in the integration and examination of three comprehensive data sources. A limitation is the lack of a more differentiated analysis of the total number of treatment cases in both the hospital and ambulatory sectors. Additionally, the analysis year 2021 was still influenced by altered patterns of service utilization due to the COVID-19 pandemic. This may have led to temporary shifts in patient behavior, such as delayed or foregone treatments, or changes in referral practices. On the supply side, healthcare providers may have reduced capacities or reorganized services, particularly in the hospital sector. These pandemic-related disruptions could have distorted usual utilization patterns and thus affected the observed associations between regional characteristics and case numbers. It should also be noted that we used GWR as an exploratory method, and only a single data point was available per district, weighted based on values from surrounding regions. As such, the results are preliminary and warrant further investigation. Future evaluations should investigate the relationship between supply density and the number of hospital and ambulatory treatment cases in relation to additional factors, such as socioeconomic inequalities or types of illnesses, as well as the adequacy of regional services concerning actual health needs [44].

## Data Availability

Two of the three datasets used and analyzed during the current study are publicly available from the Regional Database (RDB) of the Federal and State Statistical Offices and the INKAR database of the Federal Institute for Research on Building, Urban Affairs, and Spatial Development (BBSR). However, due to data protection regulations, the health insurance claims data are not publicly available.
